# Frequency-Bessel Transform Based Microtremor Survey Method and Its Engineering Application

**DOI:** 10.3390/ijerph192013484

**Published:** 2022-10-18

**Authors:** Zhiwei You, Peifen Xu, Jing Qian, Lianpeng Cao, Yanan Du, Qiang Fu

**Affiliations:** 1 Shenzhen Institute of Advanced Technology, Chinese Academy of Sciences, Shenzhen 518055, China; 2 Shenzhen Development Research Center for Natural Resource and Real Estate Assessment, Shenzhen 518034, China; 3 Key Laboratory of Shale Gas and Geoengineering, Institute of Geology and Geophysics, Chinese Academy of Sciences, Beijing 100029, China; 4 Innovation Academy of Earth Science, Chinese Academy of Sciences, Beijing 100029, China; 5 College of Earth and Planetary Sciences, University of Chinese Academy of Sciences, Beijing 100049, China; 6 Xiongan Urban Planning and Design Institute Co., Ltd., Baoding 071700, China

**Keywords:** frequency-Bessel transform, microtremor survey, inversion, genetic algorithm

## Abstract

The development and utilization of urban underground space depend heavily on an understanding of urban geological conditions. The microtremor survey method is essential in urban geological surveys due to its quickness, convenience, non-destructiveness, and interference resistance. Since only the fundamental dispersion curves of Rayleigh waves can be obtained by utilizing the spatial autocorrelation method, the inversion results have multiple solutions. To improve the accuracy of the microtremor survey, this study employed the frequency-Bessel transform to extract the fundamental and higher modes of dispersion information of Rayleigh waves from the microtremor data array and verified the effectiveness of this method by synthesizing theoretical microtremor signals. Additionally, taking into account the order identification challenges brought on by mode jumps or missing modes in the dispersion curve, this study processed a multi-mode dispersion curve based on the newly proposed inversion objective function coupled with a genetic algorithm to obtain a shallow surface S-wave velocity structure. Compared to the traditional inversion objective function, the new function presented in this study could address mode misidentification more effectively and improved the accuracy of inversion calculations. Finally, the applicability and dependability of the frequency-Bessel-transform-based microtremor survey method were evaluated in a practical engineering case.

## 1. Introduction

As China’s urbanization process continues to expand, there is a growing need for urban land. Urban underground space must be developed and used in order to promote the sustainable growth of cities and alleviate the shortage of urban land resources. Hence, it is crucial to fully comprehend urban geological conditions. The microtremor survey plays a vital role in urban geological surveys due to its advantages of having no artificial seismic source, strong resistance to electromagnetic interference, quick speed, and convenience. Research shows that microtremor signals consist of high-frequency (>1 Hz) and low-frequency (<1 Hz) signals. Low-frequency signals are primarily produced by natural events, such as ocean tides, pressure variations, and volcanic activity, whereas high-frequency signals are generally produced by human activities, such as traffic and mechanical vibration [1,2,3]. The microtremor survey method, a geophysical detection technique, employs microtremor signals to extract a Rayleigh surface wave dispersion curve. The dispersion curve is then inverted to obtain the S-wave velocity structure of the subsurface media, which serves the exploration objective [1]. The detection of buried faults [4,5,6], underground boulders [7], the soil–rock interface [8,9,10], goaf [11], and landslide mass structures [12,13] are a few of the engineering issues that have been successfully solved using the microtremor survey method in recent years.

The most crucial step in the microtremor survey method is accurately extracting the dispersion curve of a Rayleigh wave from microtremor signals since the accuracy of the dispersion curve directly affects the reliability of the inversion results. The principal techniques used nowadays for extracting dispersion curves include the spatial autocorrelation method [1,14], the extended spatial autocorrelation method [15,16], the frequency–wavenumber method [17,18], the frequency-Bessel transform method [19], etc. Notably, the fundamental Rayleigh wave energy is assumed to be dominant in the surface wave in both the spatial autocorrelation method and the extended spatial autocorrelation method, ignoring the influence of the higher mode of Rayleigh wave energy. Based on this, only the phase velocity dispersion curve of the fundamental Rayleigh wave can be obtained, which has the disadvantage of increasing the non-uniqueness of the inversion result. A precise Rayleigh wave dispersion curve cannot be extracted because some body waves included in microtremor signals may cause several peaks in the F–K dispersion spectrum. As a result, the frequency-Bessel transform (F-J transform) can extract the fundamental dispersion curve of a Rayleigh wave from microtremor signals, as well as details about the higher mode of the Rayleigh wave when compared to other techniques.

The S-wave velocity structure of the underground media is obtained by geophysical inversion. However, since geophysical inversion is a severely understudied subject, its conclusions are not reliable enough. According to the research, the joint inversion of multi-mode dispersion curves can effectively reduce the non-uniqueness and increase the accuracy of inversion outcomes [20,21]. For complicated, near-surface media, it is challenging to precisely detect the multi-mode dispersion curves in the surface wave energy spectrum. For instance, mode kissing [22,23] and leaking waves [24] may result in mode misidentification, which can compromise the accuracy of inversion results. In light of this, this study proposed a new inversion objective function to jointly inverse Rayleigh waves’ fundamental and higher modes of dispersion curves. Mode misjudgment can be prevented since the proposed inversion objective function does not have to determine the modes of the selected Rayleigh wave dispersion curves. Additionally, there is no need for the forward calculation of the dispersion curve during the inversion process, which can significantly increase inversion effectiveness.

First, this study constructs a novel inversion objective function based on the frequency-Bessel transform method and synthesizes the theoretical microtremor data of three typical geological models. Second, based on the synthesized signals, this study verifies the effectiveness of the frequency-Bessel transform method and the reliability of the new inversion objective function. Finally, using a practical engineering case, the application effect of the proposed method is verified.

## 2. Methodology

### 2.1. Frequency-Bessel (F-J) Transform

The frequency-Bessel transform method, like the spatial autocorrelation method, assumes that the sources of the microtremor wave field are randomly distributed and isotropic and that the stratum is a horizontally layered homogeneous medium. Based on this, the cross-correlation function of the vertical component of microtremor signals between two stations after the frequency-Bessel transform is as follows [25]:

(1) $$I\left(\omega ,k\right)=\int_{0}^{+\infty }C\left(r,\omega \right)J_{0}\left(kr\right)rd_{r},$$

where C(r,ω) is the cross-correlation function of the vertical component of microtremor signals between stations, $J_{0}$ represents the zero-order Bessel function, k refers to the wavenumber, r is the distance between the two stations, and ω is the angular frequency.

The research shows that the Fourier transform of the cross-correlation function of microtremor signals between two points in space is proportional to the imaginary part of its Green function, and there is only a difference in amplitude [26], that is:

(2) $$C\left(r,\omega \right)=A\cdot Im\left\{G_{zz}\left(r,z=0;\omega \right)\right\},$$

where A is a constant, $G_{zz}\left(r,z=0;\omega \right)$ is the vertical component of Green’s function in the frequency domain, and the definition of z=0 is that all observation stations are on the surface. For half-space horizontally layered elastic media with isotropic sources (such as point sources and explosion sources of vertical vibration), the Green’s function can be written as follows [27,28]:

(3) $$G_{zz}\left(r,z=0;\omega \right)=\int_{0}^{+\infty }g_{z}\left(z=0,\kappa ,\omega \right)J_{0}\left(\kappa r\right)\kappa d_{\kappa },$$

where $g_{z}\left(z=0,\kappa ,\omega \right)$ is the kernel function of the Green function, which is only related to wave number *k* and angular frequency ω, and has nothing to do with the distance r.

According to the orthogonality of the Bessel function, Formula (4) can be obtained:

(4) $$\int_{0}^{+\infty }J_{0}\left(kr\right)\cdot J_{0}\left(\kappa r\right)rd_{r}=\frac{1}{\sqrt{\kappa k}}\delta \left(\kappa -k\right),$$

Equations (2)–(4) can be substituted into Equation (1) and simplified to obtain Equation (5):

(5) $$I\left(\omega ,k\right)=A\cdot Im\left\{g_{z}\left(z=0,k,\omega \right)\right\}.$$

Since the wavenumber k=ω/c=2πf/c, *c* is the phase velocity, f is the frequency, and Equation (5) can also be written as follows:

(6) $$I\left(f,c\right)=A\cdot Im\left\{g_{z}\left(z=0,f,c\right)\right\}.$$

Additionally, since the kernel function of a Rayleigh wave’s Green function has frequency dispersion [27], I(ω,k) also presents dispersion characteristics. When the appropriate frequency range and phase velocity range are given, a series of I(f,c) or I(ω,k) values can be calculated according to Formula (1), and the formed two-dimensional image is called a Rayleigh wave dispersion diagram.

### 2.2. Construction of New Inversion Objective Function

The forward modeling of a dispersion curve is the basis of its inversion. As for the calculation of the Rayleigh wave dispersion curve of a horizontally layered media model, the generalized reflection–transmission coefficient method [29] is frequently preferred because it is more stable and accurate in the high-frequency band than other methods. However, for a medium model with low-velocity layers, using a single secular function to search the root leads to the problem of leaky roots. He Yaofeng successfully solved this problem by introducing the concept of the secular function family, which can be stated as follows [30]:

(7) $$det\left\{I-{\hat{R}}_{ud}^{\left(j-1\right)}{\hat{R}}_{du}^{\left(j\right)}\right\}=0,\;j=1,2,\ldots ,\mathrm{Nt}-1,\;det\left\{E_{21}^{\left(1\right)}+E_{22}^{\left(1\right)}\Lambda_{u}^{\left(0\right)}{\hat{R}}_{du}^{\left(1\right)}\right\}=0,$$

where Nt represents the number of layers of the medium model, and the definitions of other variables can be referred to in Chen (1993) [29]. For any given frequency, one can always find a root or a series of roots that satisfy any equation in the secular function family, and these roots are the phase velocities of Rayleigh waves.

The traditional inversion objective function [31,32] is to fit the measured dispersion curve with the theoretical dispersion curve calculated by the model to minimize the fitting difference. The traditional inversion objective function can be expressed as follows [32]:

(8) $$E\left(m\right)={\left\{\sum_{i=1}^{N}{\left[w_{i}\cdot \left|c_{i}^{obs}-g\left(f_{i}^{obs},m\right)\right|\right]}^{l}\right\}}^{1/l},$$

where E(m) is the fitting difference of the geological model m, N is the number of dispersion points, $w_{i}$ is the weight factor of the dispersion point i, $c_{i}^{obs}$ is the measured value corresponding to the frequency point $f_{i}^{obs}$, m represents the theoretical geological model, $g\left(f_{i}^{obs},m\right)$ represents the theoretical phase velocity value of the frequency point $f_{i}^{obs}$ of model m, and l is the norm, which is generally set at 2.

According to the concept of secular function family, any given dispersion point $\left(f,V_{ph}\right)$ can be substituted into the secular function family corresponding to the model. If the minimum value of the N function tends toward 0, $V_{ph}$ can be considered as the Rayleigh wave phase velocity of the model at frequency f. Hence, the expressions that define $S_{0}\left(f,V_{ph}\right)$ and $S_{j}\left(f,V_{ph}\right)$ are as follows:

(9) $$S_{0}\left(f,V_{ph}\right)=det\left\{E_{21}^{\left(1\right)}+E_{22}^{\left(1\right)}\Lambda_{u}^{\left(0\right)}{\hat{R}}_{du}^{\left(1\right)}\right\},$$

(10) $$S_{j}\left(f,V_{ph}\right)=det\left\{I-{\hat{R}}_{ud}^{\left(j-1\right)}{\hat{R}}_{du}^{\left(j\right)}\right\},\;j=1,2,\ldots ,N-1.$$

For measured data point $\left(f^{obs},c^{obs}\right)$, the secular function value is defined as:

(11) $$S\left(f^{obs},c^{obs}\right)=min\left\{S_{0}\left(f^{obs},c^{obs}\right),S_{1}\left(f^{obs},c^{obs}\right)\ldots S_{N-1}\left(f^{obs},c^{obs}\right)\right\}.$$

Therefore, this study proposed a new inversion objective function, which is expressed as follows:

(12) $$E\left(m\right)={\left\{\sum_{i=1}^{N}{\left[w_{i}\cdot \left|S\left(f^{obs},c^{obs},m\right)\right|\right]}^{l}\right\}}^{1/l},$$

where E(m) is the fitting error of the geological mode m, N is the number of dispersion points, $w_{i}$ is the weight factor of the dispersion point i, $c^{obs}$ is the measured value corresponding to the frequency point $f^{obs}$, and m represents the theoretical geological model. $S\left(f^{obs},c^{obs},m\right)$ is the secular function value at the measured data point $S\left(f^{obs},c^{obs}\right)$ when the model is m. The new inversion objective function does not need to calculate the theoretical phase velocity corresponding to the frequency $f^{obs}$ under the model m, avoiding the root-searching process and improving the calculation efficiency of the inversion.

## 3. Numerical Tests

To assess the validity of the proposed inversion objective function, this study employed a modal superposition algorithm [33] to synthesize the theoretical microtremor data of three typical geological models. Then, based on the frequency-Bessel transform method, the synthetic microtremor signals were processed, and the extracted dispersion curve was inverted using the new inversion objective function. Finally, this study’s investigation was concluded by contrasting the inversion structure with the actual model.

### 3.1. Selection of Geological Model

In a near-surface geophysical investigation, three typical geological models are usually encountered, namely, the velocity increase model, the low-speed soft interlayer model, and the high-speed hard interlayer model [34]. When the stratigraphic sedimentary environment is relatively stable, the stratigraphic structure adheres to the velocity increase model. The stratigraphic structure can be described by the low-speed soft interlayer model when there are unfavorable geological bodies, such as karst and fault fracture zones, in the subsurface medium because the medium is loose and rich in water. The high-speed hard interlayer model can be used to describe a stratigraphic structure with unfavorable geological bodies, such as boulders and intrusive rock masses. These three typical geological models were chosen for this study’s synthesis of theoretical microtremor signals because they are widely representative, and their parameters are provided in Table 1. The three models were all four-layer geological models in which the S-wave velocity of the velocity increase model (Model 1) increased as depth grew. The low-speed soft interlayer model (Model 2) and the high-speed hard interlayer model (Model 3) had a soft interlayer with an S-wave velocity of 150 m/s and a hard interlayer with an S-wave velocity of 350 m/s in the second layer, respectively.

### 3.2. Theoretical Synthesis of Microtremor Signals

**Figure 1:**
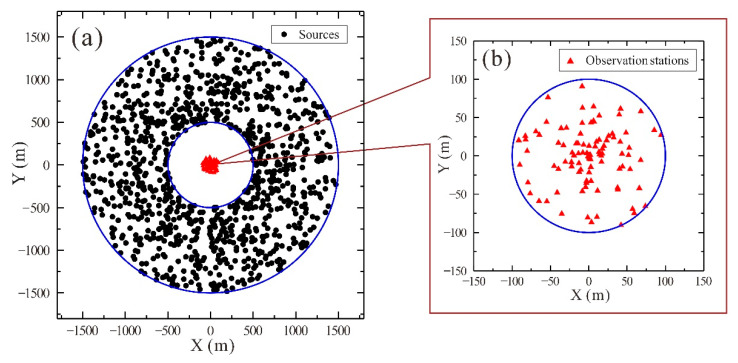
Spatial distribution diagram of random sources and observation array: (**a**) random sources; (**b**) observation array.

**Figure 2:**
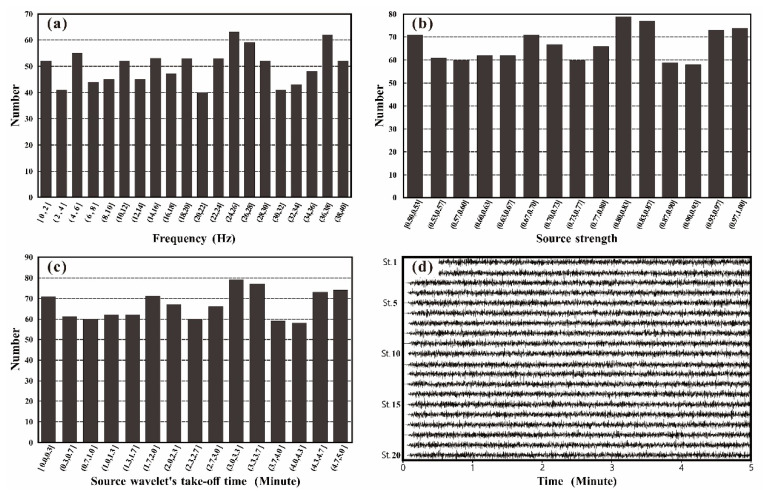
Theoretical synthesis of microtremor signals: (**a**) histogram of dominant frequency distribution of sinusoidal exponential decay wavelet; (**b**) source strength distribution histogram; (**c**) distribution histogram of source wavelet take-off time; (**d**) waveform diagram of microtremor signals of 20 stations synthesized by model 1.

There is a minimum required number of stations since a finite discrete summation is required to substitute the integral operation (Equation (1)) when obtaining a Rayleigh wave dispersion diagram using the frequency-Bessel transform method. A total of 100 observation stations were randomly distributed within a 100 m radius of the site to synthesize theoretical microtremor signals (Figure 1b). Additionally, 1000 sources in the vertical direction were randomly distributed within a 500–1500 m radius of the site (Figure 1a). The sources’ time function was a sinusoidal exponential decay wavelet, with a source strength that was randomly generated between 0.5 and 1 (Figure 2b) and a dominant frequency that was randomly distributed between 0.01 Hz and 40 Hz (Figure 2a).

Observation stations and sources were distributed on the surface, and the take-off of the source wavelet occurred at random between 0 and 5 min (Figure 2c). In this study, a modal superposition algorithm [33,35] was employed to calculate the theoretical microtremor signals of the vertical component with a synthetic sampling frequency of 100 Hz and a time length of 5 min (Figure 2d).

### 3.3. Microtremor Signal Processing

**Figure 3:**
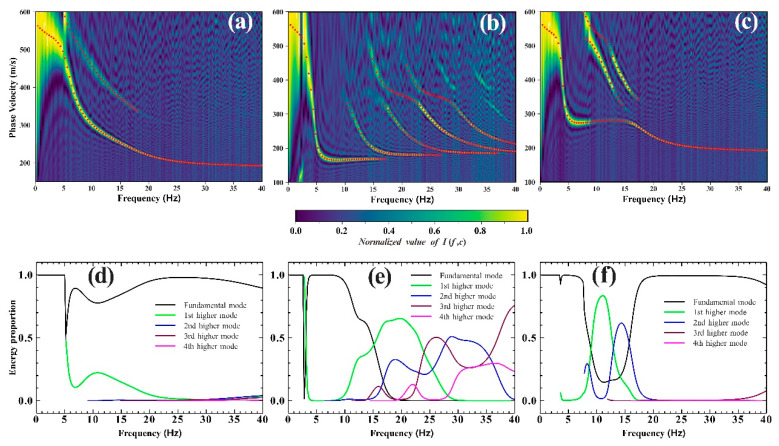
The Rayleigh wave dispersion diagram extracted from the synthesized microtremor signals and the energy proportions of the fundamental and the first three high-order Rayleigh waves corresponding to the three geological models. Red dots and lines represent the dispersion curves of fundamental and higher-order Rayleigh waves calculated theoretically. (**a**,**d**) Model 1; (**b**,**e**) Model 2; (**c**,**f**) Model 3.

In this study, the synthetic theoretical microtremor signals were processed using the frequency-Bessel transform method. The computed frequency range must be supplied to calculate the Rayleigh wave dispersion diagram. Since the dominant frequency of the source wavelet was randomly distributed in the range of 0.01–40 Hz, this paper only calculated a Rayleigh wave dispersion diagram with the frequency range of 0.2–40 Hz. The stations in this study were first paired, and the cross-correlation functions of each group of stations were then determined. Then, the frequency-Bessel transform was applied to the resulting cross-correlation function over an acceptable range of phase velocity variation using Formula (1). Finally, the Rayleigh wave dispersion diagrams of the three geological models could be obtained, as shown in Figure 3a–c.

To verify the effectiveness of the frequency-Bessel transform, this study employed the generalized reflection-transmission coefficient method to calculate the fundamental and higher modes of the Rayleigh wave dispersion curves of the three geological models. Then, the calculated multi-mode dispersion curves were superimposed on the corresponding Rayleigh wave dispersion diagrams. According to Figure 3a–c, the peak distribution of the Rayleigh wave dispersion diagram corresponding to the three geological models was in good agreement with the dispersion curve obtained through theoretical calculation. However, the dispersion diagram showed instances of missing modes and mode jumps across several frequency bands. For instance, the fundamental dispersion curve of Model 3 was absent between 8 Hz and 16 Hz, whereas the high-mode dispersion curve was present in the ranges of 8–13 Hz and 13–16 Hz. Therefore, without further information, it was difficult to accurately determine the order of the dispersion curves.

The fraction of Rayleigh wave energy in each mode can be used to explain why the dispersion curve in some frequency bands of the Rayleigh wave dispersion diagram was absent. In an analysis of multi-mode Rayleigh wave signals, Tokimatsu [36] initially developed a dielectric response function and provided a method for calculating the percentage of multi-mode Rayleigh wave energy. The calculation results of the energy proportion for Models 1, 2, and 3 of the Rayleigh waves are shown in Figure 3d–f, respectively, only considering the fundamental and first four high modes. The energy distribution diagram makes it clear that the presence of each mode’s dispersion curve in the dispersion diagram depended strongly on the energy proportion of each mode’s Rayleigh wave. If the energy proportion was large, there was a dispersion curve in the dispersion diagram; otherwise, there was not one.

**Figure 4:**
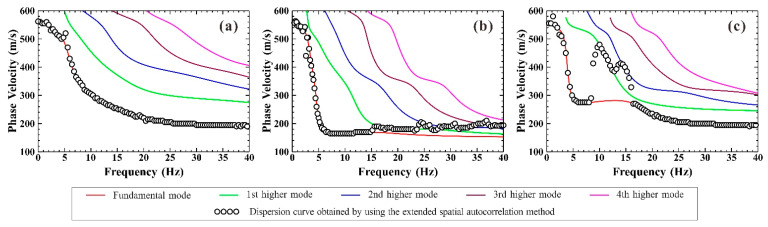
The Rayleigh wave dispersion curves obtained based on the extended spatial autocorrelation method and the theoretical calculation of the fundamental and first three high-order Rayleigh waves corresponding to the three geological models. (**a**) Model 1; (**b**) Model 2; (**c**) Model 3.

To demonstrate the superiority of the frequency-Bessel transform method, the extended spatial autocorrelation method was supplemented to process the synthetic theoretical microtremor signals. Similarly, this paper calculated the Rayleigh wave dispersion curve for the frequency range of 0.2–40 Hz. As shown in Figure 4, for each geological model, only one dispersion curve of Rayleigh wave was obtained using the extended spatial autocorrelation method. Meanwhile, for Model 2 and Model 3, the dispersion curves obtained were quite different from the theoretical fundamental dispersion curves. Combined with Figure 3d–f, the dispersion curves obtained based on the extended spatial autocorrelation method were the result of the superposition and coupling of the fundamental Rayleigh wave and the higher-mode Rayleigh waves [36].

### 3.4. Inversion of Multi-Mode Dispersion Curve

Rayleigh wave dispersion curve inversion is a highly nonlinear optimization problem with multiple extreme values and parameters, and the local linearization inversion method is highly dependent on the initial model. Hence, a genetic algorithm was employed in this study to inverse the dispersion curve. A large number of studies have demonstrated that the sensitivity of surface wave dispersion to layer thickness and S-wave velocity is much higher than that of P-wave velocity and medium density [15]. Therefore, only layer thickness and S-wave velocity were inversed in this study, while the P-wave velocity and medium density could be determined by the empirical relationship between them and S-wave velocity [15].

**Figure 5:**
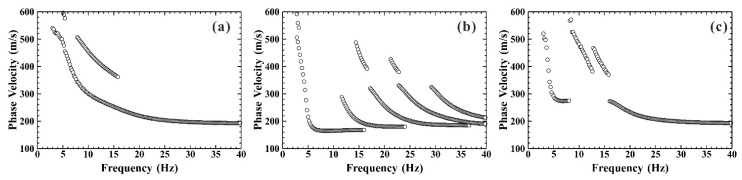
The dispersion curves detected from Rayleigh wave dispersion diagram. (**a**) Model 1; (**b**) Model 2; (**c**) Model 3.

In this study, the multi-mode dispersion curves of Rayleigh waves were computed using both the conventional and newly proposed inversion objective functions, and the benefits and drawbacks of the two approaches were assessed by contrasting their inversion outcomes and calculation durations. Based on the Rayleigh wave dispersion diagrams of the three geological models (Model 1, Model 2, and Model 3) obtained by processing synthetic microtremor signals in the previous section, this study detected the multi-mode dispersion curves of Rayleigh waves through programming according to the background values of the images (shown in Figure 5). Meanwhile, it was assumed that the order of the multi-mode dispersion curves was correctly identified according to the prior information. To ensure the objectivity of the comparison in terms of the computation times based on different inversion objective functions, the input parameters of the genetic algorithm utilized in the inversion calculation were the same, with the calculation program running on the same Dell 7050 Tower 006348 desktop computer. The inversion adopted a four-layer geological model, and the search ranges of the S-wave velocity and each layer’s thickness are shown in Table 2.

**Figure 6:**
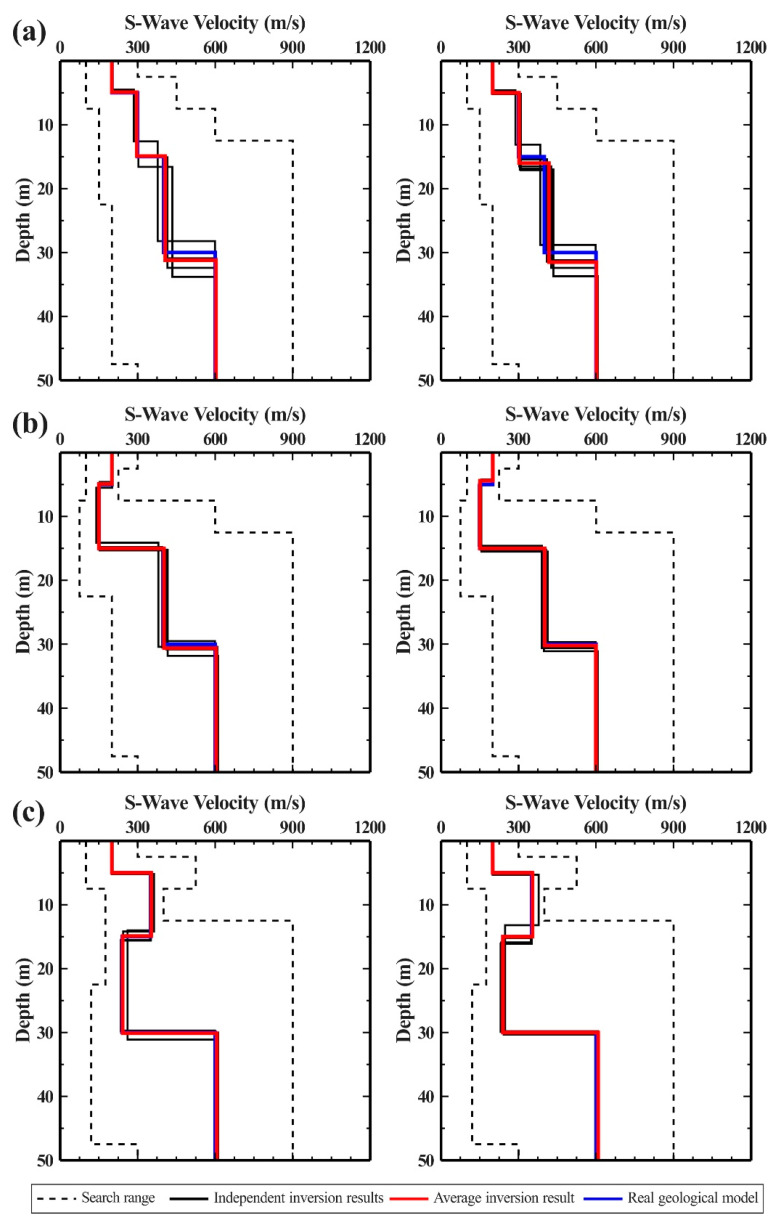
Inversion results of multi-order dispersion curves. The left and right figures correspond to the traditional and newly proposed inversion objective functions, respectively. (**a**) Model 1; (**b**) Model 2; (**c**) Model 3.

Six separate inversion calculations were performed for each of the three models to assess the stability of the inversion algorithm. The six results were then superimposed and averaged to provide the inversion results. Figure 6 displays the inversion results of the dispersion curves of the three models using different objective functions, with the left ones based on the traditional function and the right ones based on the newly proposed function. The results suggest that the inversion results were highly congruent with the actual geological model, regardless of whether the traditional or newly proposed functions were applied. Additionally, there were not many significant differences between the outcomes of each separate inversion. The reason is that the number of possible inversion solutions could be considerably reduced by jointly inverting the fundamental and higher-order dispersion curves.

To illustrate the benefits and drawbacks of the inversion algorithm more clearly, this study performed an error analysis on the inversion results (Table 3). The results demonstrate that, after superimposing and averaging the inversion results, the relative error was less than 10% compared to the actual model, validating the efficacy and application of the inversion algorithm based on the newly proposed function. Although both the inversion objective functions could reconstruct the actual stratum structure, the traditional function necessitated accurate prior knowledge of the mode of each order of dispersion curve. Table 4 summarizes the times of each inversion calculation, indicating that the inversion calculation time based on the newly proposed function was significantly less than that of the traditional function. The primary reason for this obvious distinction is that the traditional inversion Objective function must first perform the forward calculation of the multi-mode dispersion curve. This process necessitated frequent calls to the secular function family for root calculation, which directly increased the algorithm’s overall calculation time. Instead of requiring forward computation, the newly proposed function could greatly reduce calculation time and improve inversion calculation efficiency without compromising the accuracy and stability of the inversion outcomes.

## 4. An Engineering Application Verification

This study carried out application research in Guangzhou with the purpose of testing that the frequency-Bessel transform method based on the newly proposed objective function could extract high-mode Rayleigh waves from microtremor signals and obtain the S-wave velocity structure of a high-precision shallow surface through inversion.

### 4.1. Site Overview

**Figure 7:**
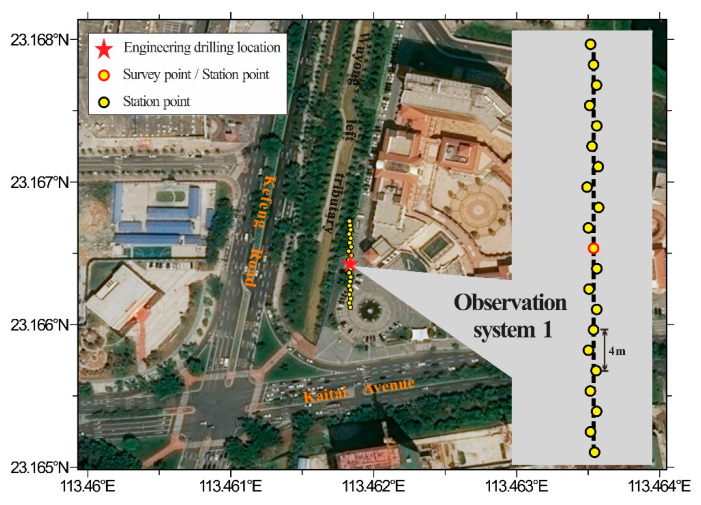
Schematic diagram of the test site and engineering drilling location.

The experimental site was located next to a project’s drilling hole at the DOPO International Plaza in Huangpu District, Guangzhou (Figure 7). The site was flat and open, making it suitable for data acquisition. As a part of the South China paraplatform, Guangzhou straddles the two major tectonic units of central and northern Guangdong. This region sees frequent crustal activity through neotectonic movement, forming different geological structures, such as fold zones, fault zones, and inland fault basins [37]. A geotechnical investigation report suggested that the site’s upper part of its quaternary formation (Q4) was Qml and Qpal, and the underlying bedrock was late Silurian granite. Additionally, according to the weathering degree of the rock, the site was divided into completely weathered, strongly weathered, and moderately weathered zones.

### 4.2. Data Acquisition

Because the amplitude of the microtremor signals was very weak and the frequency band was wide, the precision and sensitivity of the observation instruments were required to be high. Some independent microtremor detectors were used for data acquisition in this engineering application. Each microtremor detector system mainly included a geophone with a natural frequency of 2 Hz and a data logger of the model Datamark LS-8800. The main performance indicators of the geophone and recorder are shown in Table 5.

**Figure 8:**
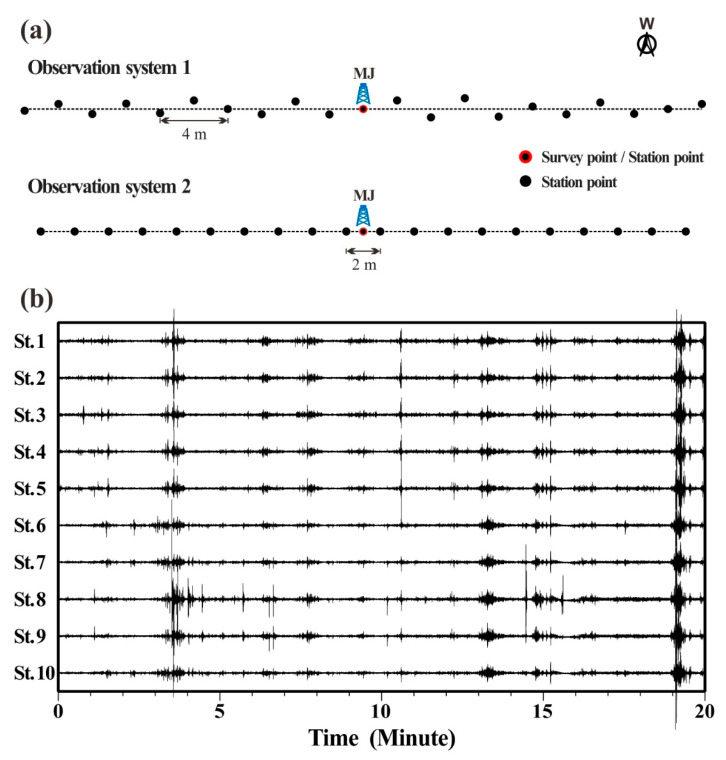
Waveform of observation array and part of measured microtremor signals. (**a**) Schematic diagram of observation array; (**b**) waveforms of microtremor signals at part of observation stations.

**Figure 9:**
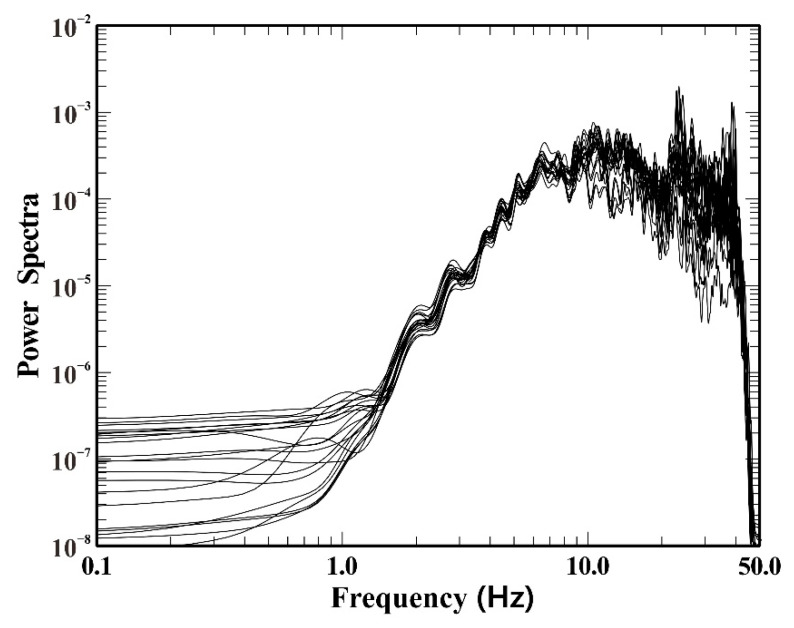
Power spectra of microtremor signals recorded by the corresponding stations of observation system 1.

Since there was a parking lot close by, only the hour at midday when there were few vehicles was chosen for monitoring for safety concerns. Compared to the randomly distributed observation array, a linear or nearly linear observation array is more convenient to deploy and easier for determining the detection point. Therefore, linear and nearly linear observation arrays were employed to collect microtremor signals in this engineering application. The observation system is depicted in Figure 8a. A total of 21 stations made up observation system 1, which was roughly linear but had inconsistent distances between adjacent stations. For observation system 1, the minimum distance between pairs of stations was 2.02 m and the maximum was 40.5 m. Observation system 2 was linear, including 21 stations, and there were 1 or 2 m between each station. Obviously, the minimum distance between pairs of stations was 1 m, and the maximum was 38 m for observation system 2. Moreover, the number of station pairs with different radii in observatory array 1 was 71, while the number in observatory array 2 was only 30. Two independent observations using these two systems were conducted, each lasting 20 min and using a sampling frequency of 100 Hz. Figure 8b depicts the waveforms of microtremor signals from some observation stations corresponding to observation system 1. All observation instruments were of the same batch and model, which made it unnecessary to remove the instrument response. Figure 9 displays the power spectra of the microtremor signals obtained through the smoothing process of the Parzen window, indicating an intuitive analysis of the energy of the measured microtremor signals. The frequency band of 5–40 Hz was where the strongest energy was present in the power spectra of 21 stations.

### 4.3. Measured Microtremor Signal Processing

The measured microtremor data must first undergo preprocessing, which included the removal of instantaneous interference, de-meaning, de-trending, Butterworth band-pass filtering, time–domain normalization, and frequency–domain normalization. Then, the Rayleigh wave dispersion diagram was extracted from the microtremor data array using the frequency-Bessel transform method in accordance with the theoretical derivation in Section 2.1. The specific steps are summarized as follows: (1) Segmentation. Divide the microtremor data that eliminate interference signals into 30 segments, each with a length of 20.48 s, and partial overlapping in adjacent data segments. (2) Calculate the cross-correlation function. Pair the stations in the array, and calculate each pair’s cross-correlation function and station spacing. Then, superimpose and average the cross-correlation functions of station pairs with equal spacing in the array to obtain a cross-correlation function with different station spacing. (3) Smoothing. Utilize the Parzen window to smooth the cross-correlation function. (4) Transform application. The frequency-Bessel transform method is carried out on the cross-correlation function according to Formula (1). The integration is replaced with the finite discrete summation, and the Rayleigh wave’s dispersion diagram is obtained.

**Figure 10:**
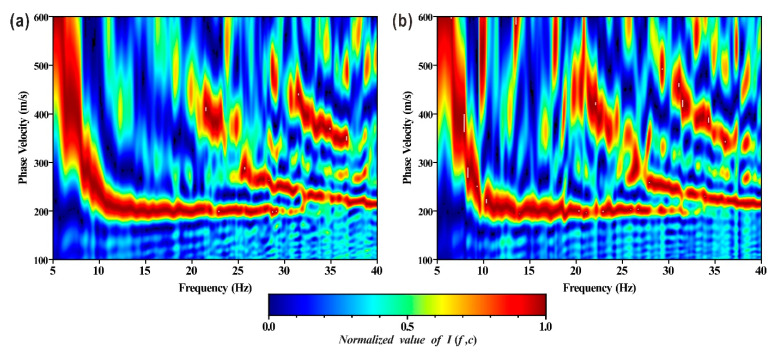
Rayleigh wave dispersion diagram extracted from measured array microtremor data using frequency-Bessel transform method. (**a**) Observation system 1; (**b**) observation system 2.

The measured microtremor data collected by the two observation systems were processed based on the above steps. The calculation results are shown in Figure 10. Similarities between Figure 10a,b included the presence of distinct fundamental and high-order dispersion curves of multiple modes, as well as the energy of the fundamental Rayleigh wave being stronger in the low-frequency part, whereas the energy of the high-mode Rayleigh wave was dominant in the high-frequency part. The difference was that the dispersion curve in Figure 10a was more continuous and clear at low frequencies, and the peak area was likewise more concentrated with high resolution. In general, the imaging quality of the dispersion diagram obtained with observation system 1 was superior to that of system 2 because system 1 could obtain more sample values of the cross-correlation function, which was more conducive to the superposition and summation of finite discretions.

**Figure 11:**
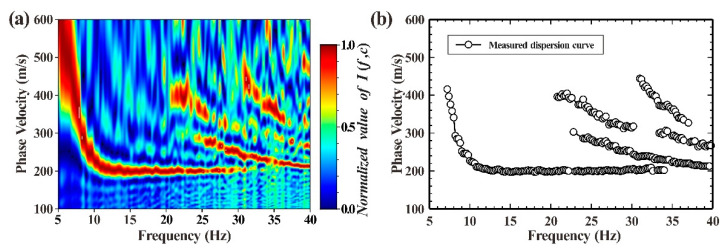
Dispersion diagram and detected measured dispersion curve obtained using frequency-Bessel transform after the combination of cross-correlation functions calculated by the two observation systems. (**a**) The dispersion diagram; (**b**) The detected measured dispersion curve.

The station spacing and frequency were the only factors that affected the cross-correlation function C(r,ω) for the same stratigraphic structure. Using various observation arrays for repeated observations allowed for the collection of more cross-correlation function sample values, which also produced a dispersion diagram with an improved imaging effect. Therefore, this study combined the cross-correlation functions calculated by the two observation systems, followed by the frequency-Bessel transform. The results are shown in Figure 11a. Figure 11a displays a higher resolution at low frequencies and smoother dispersion curves when compared to Figure 10a. Therefore, when the number of field observation stations was limited, alternative observation systems could be employed for numerous observations to improve detection results. Multiple observations on the same detection point, on the other hand, increased the time cost of data acquisition, so all factors should be considered comprehensively for the best acquisition scheme.

**Figure 12:**
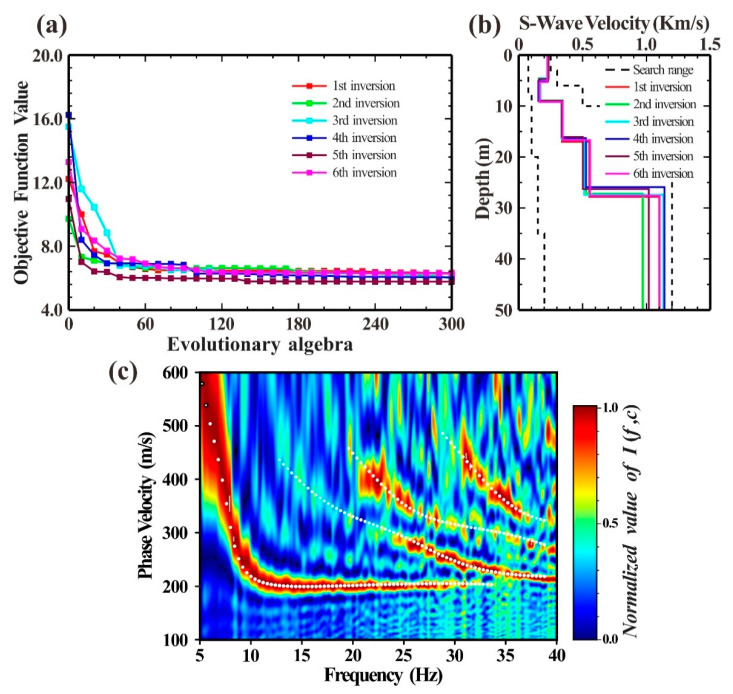
Joint inversion results of fundamental and higher-order dispersion curves. (**a**) Curve of objective function value; (**b**) inverse S-wave velocity structure; (**c**) comparison between the theoretical dispersion curve corresponding to the first inversion result and the measured dispersion diagram. The white dotted line is the fundamental and higher-order dispersion curve of the theoretical calculation.

Figure 11b displays the multi-mode dispersion curve of the Rayleigh wave picked up from Figure 11a. As can be observed, it was simple to identify the fundamental mode of the dispersion curve, but it was more difficult and prone to misidentification to identify the higher modes without any prior information. Therefore, this study proposed a new inversion objective function based on a genetic algorithm to process the selected multi-order dispersion curve of the Rayleigh wave. Figure 12 displays the outcomes of six independent inversions of the selected fundamental and higher-mode dispersion curves. The six results pointed to a good consistency between the objective function change curve and the S-wave velocity structure obtained by inversion, as well as the presence of a clear, low-speed interlayer in the second layer, which suggests that the inversion’s multiple solutions could be minimized by combining the inversion of fundamental and high-mode dispersion curves.

**Figure 13:**
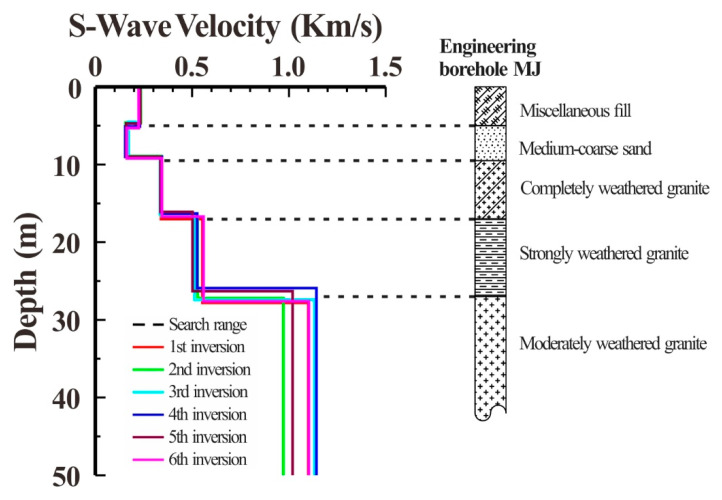
Comparison between inversion results and borehole histogram.

As illustrated in Figure 8a, there was an engineering borehole MJ close to the detection spot. To verify the accuracy of the detection results, the stratigraphic structure information obtained by inversion could be compared with the borehole information. According to the results of six independent inversions in Figure 13 and the borehole histogram, the inversion results were fairly accurate representations of the actual stratigraphic structure. Additionally, the low-speed soft interlayer, which corresponded to the medium-sand seam in the borehole histogram, was well-characterized in the observation point’s underlying structure. The detection results supported the frequency-Bessel transform method’s ability to successfully extract the multi-order dispersion curve of the Rayleigh wave from the microtremor data array. It also demonstrated that the newly proposed inversion objective function can effectively utilize both fundamental and high-mode Rayleigh wave information while preventing mode misidentification and improving the accuracy of the microtremor survey.

## 5. Conclusions

The frequency-Bessel transform-based microtremor survey method was methodically and thoroughly investigated in this study. Through the numerical modeling of hypothetical microtremor signals of three typical geological models, the efficacy and validity of this method for extracting the fundamental and high-mode dispersion information of Rayleigh waves from a microtremor data array were verified. This study also proposed a new objective function for inversion, which improved the calculation efficiency of the inversion algorithm compared to the traditional one since no forward calculation of the dispersion curve was required. Additionally, the newly proposed inversion objective function did not need to identify the mode of the dispersion curve, avoiding incorrect inversion results caused by misidentification.

According to engineering application research in Guangzhou, the frequency-Bessel transform method could efficiently extract the fundamental and high-mode dispersion information of Rayleigh waves from measured microtremor signals and could produce a more precise S-wave velocity structure of underground media through the joint inversion of the fundamental and high-mode dispersion curves. As a result, the frequency-Bessel-transform-based microtremor survey method was complete and feasible theoretically with a satisfactory application effect, indicating good engineering application value in urban geological surveys.

## Figures and Tables

**Table 1:** Parameters of three geological models.

Layer Serial Number	Layer Thickness (m)	Model 1			Model 2			Model 3		
		V1	V2	*ρ*	V1	V2	*ρ*	V1	V2	*ρ*
1	5	1611	200	1.725	1611	200	1.725	1611	200	1.725
2	10	1695	300	1.784	1582	150	1.697	1743	350	1.809
3	15	1798	400	1.834	1798	400	1.834	1641	240	1.750
4	∞	1969	600	1.920	1969	600	1.920	1969	600	1.920

V1 is the velocity of the P wave (m∙s^−1^), V2 is the S-wave velocity (m∙s^−1^), and *ρ* is the density (kg∙m^−3^).

**Table 2:** Search intervals of S-wave velocity and layer thickness during inversion.

Layer Serial Number	Search Interval of Layer Thickness (m)	Search Interval of S-Wave Velocity (m/s)		
		Model 1	Model 2	Model 3
1	2.5~7.5	100~300	100~300	100~300
2	5~15	150~450	75~225	175~525
3	5~25	200~600	200~600	120~400
4	∞	300~900	300~900	300~900

**Table 3:** Relative errors between the results of different inversion objective functions and the real model.

Layer Serial Number	Model 1				Model 2				Model 3			
	Err1 (%)	Err2 (%)	Err3 (%)	Err4 (%)	Err1 (%)	Err2 (%)	Err3 (%)	Err4 (%)	Err1 (%)	Err2 (%)	Err3 (%)	Err4 (%)
1	2.67	0.17	0.33	0.08	3	0.08	6.33	0.25	0.43	0.26	0.33	0.24
2	0.17	1.17	9.8	0.78	1	0.22	3.33	0.44	0.67	0.81	0.75	1.24
3	8.33	1.58	3.22	4.42	3.67	0.46	1.44	0.25	1.44	0.83	0.56	1.14
4	---	0.5	---	0.31	---	0.61	---	0.06	---	1.00	---	1.36

Err1 and Err2 are the relative errors between each layer thickness and the S-wave velocity and real model in the inversion results using the conventional inversion objective function, respectively; Err3 and Err4 are the relative errors between each layer thickness and the S-wave velocity and real model in the inversion results using the newly proposed inversion objective function, respectively.

**Table 4:** Calculation time of inversion multi-order dispersion curves based on different inversion objective functions.

Inversion Serial Number	Calculation Time of Traditional Inversion Objective Function (s)			Calculation Time of Newly Proposed Inversion Objective Function (s)		
	Model 1	Model 2	Model 3	Model 1	Model 2	Model 3
1	6562.88	8963.45	6489.65	69.17	83.94	66.05
2	6462.36	9032.13	6875.24	67.81	83.30	66.08
3	6655.34	9268.64	6259.36	60.73	83.55	65.47
4	6034.07	9149.32	6387.68	61.58	83.70	64.94
5	6243.52	8989.64	6512.35	60.41	86.88	64.25
6	6368.43	9432.61	6659.84	62.19	86.36	65.06

**Table 5:** The main performance indicators of independent microtremor detectors.

Name	Model	Main Performance Indicators
Geophone	CDJ-S2C	Velocity type:
		three-component
		Natural frequency: 2 ± 10% Hz
		Voltage output sensitivity: 2 ± 10% V/cm/s
		Coil resistance: 6040 ± 5% KΩ
		Damping coefficient: 0.7 ± 10%
Data Logger	LS-8800	Number of data channels: 3
		A/D conversion: 24 bits
		Sampling frequency: 100 Hz or 200 Hz
		Dynamic range: 128 dB
		Time correction: real-time GPS automatic correction
		Applicable temperature: −20~+50 °C
